# Computational Screening and Experimental Validation of Inhibitor Targeting the Complex Formation of Grb14 and Insulin Receptor

**DOI:** 10.3390/molecules29010198

**Published:** 2023-12-29

**Authors:** Yosuke Ochi, Takanori Matsui, Keitaro Inoue, Kohei Monobe, Hiroshi Sakamoto, Shunsuke Aoki, Junichi Taira

**Affiliations:** 1 Department of Bioscience and Bioinformatics, Graduate School of Computer Science and Systems Engineering, Kyushu Institute of Technology, Iizuka 820-8502, Japan; 2 Department of Pathophysiology and Therapeutics of Diabetic Vascular Complications, Kurume University School of Medicine, Kurume 830-0011, Japan

**Keywords:** DOCK program, GOLD program, Grb14, insulin receptor (IR), structure-based drug screening (SBDS)

## Abstract

The development of drugs targeting gene products associated with insulin resistance holds the potential to enhance our understanding of type 2 diabetes mellitus (T2DM). The virtual screening, based on a three-dimensional (3D) protein structure, is a potential technique to accelerate the development of molecular target drugs. Among the targets implicated in insulin resistance, the genetic characterization and protein function of Grb14 have been clarified without contradiction. The *Grb14* gene displays significant variations in T2DM, and its gene product is known to inhibit the function of the insulin receptor (IR) by directly binding to the tyrosine kinase domain. In the present study, a virtual screening, based on a 3D structure of the IR tyrosine kinase domain (IRβ) in complex with part of Grb14, was conducted to find compounds that can disrupt the complex formation between Grb14 and IRβ. First, ten compounds were selected from 154,118 compounds via hierarchical in silico structure-based drug screening, composed of grid docking-based and genetic algorithm-based programs. The experimental validations suggested that the one compound can affect the blood glucose level. The molecular dynamics simulations and co-immunoprecipitation analysis showed that the compound did not completely suppress the protein–protein interaction between Grb14 and IR, though competitively bound to IR with the tyrosine kinase pseudosubstrate region in Grb14.

## 1. Introduction

Central mechanisms in type 2 diabetes mellitus (T2DM) are considered to be largely due to functional disruptions of gene products involved in insulin resistance [1,2,3]. The discovery of compounds targeting for such gene products is expected to be useful for understanding insulin resistance, and potential therapeutic candidates for T2DM may also emerge. Pharmaceutical agents that intervene in insulin signaling pathways are anticipated to enhance insulin sensitivity and the duration of action. However, the development of such novel drugs is associated with significant costs. Therefore, efficient techniques and methods that enable the rapid and accurate exploration of compounds from extensive chemical libraries have been eagerly awaited [4,5]. The utilization of in silico virtual screening has been anticipated as one solution to address the issues. Structure-based drug screening (SBDS), which is based on the three-dimensional structural data of target proteins, has been employed for the selection of compounds interacting with the target protein [6]. Here, we attempted to screen small compounds that affect the function of the growth factor receptor-bound protein 14 (Grb14), known to exert a negative regulatory effect on insulin signaling, using hierarchical in silico SBDS.

Grb14, which belongs to the Grb7 family of proteins (Grb7/10/14), was initially cloned from human breast epithelial cell cDNA [7]. Grb14 physically associates with the intracellular kinase domain of the insulin receptor (IR), known as a beta-subunit (IRβ), in response to insulin stimulation [8]. The IRβ–Grb14 complex formation results in the inhibition of downstream signal transduction by preventing the activation of the insulin receptor substrate-1 (IRS-1) [3,9,10], and consequently, the glucose uptake is impaired. Currently, Grb14 is believed to play a critical role in regulating insulin signaling and glucose homeostasis: (i) Grb14 knock-out mice (*Grb14*^−/−^) exhibited improved glucose tolerance, albeit lower circulating insulin levels [11]; (ii) Grb14 mRNA levels were found to be increased in adipose tissue of *ob*/*ob*, T2DM model mice [12]; (iii) genome-wide association studies for the South Asian region demonstrated a relevance of Grb14 to metabolic disorders, especially insulin resistance [13]. Grb14 has also been shown to regulate the function of the leptin receptor, which plays a critical role in regulating body weight and energy metabolism [14]. In terms of structural features, the Grb7 family of proteins is characterized by having four structural modules [8,15,16]: the RA (Ras-associating) domain, the PH (pleckstrin homology) domain, the SH2 (Src homology 2) domain, and the BPS (between the PH and SH2 domains) region. Among them, the BPS region is a unique structural feature for the Grb7 family of proteins, and the Grb14 (BPS) is primarily responsible for binding to the activated IRβ [8,15]. In fact, the crystal structure of the complex of IRβ and part of the Grb14 (BPS) (residues 373–409) was determined at a 3.2 Å resolution by Depetris et al. (PDBid: 2AUH) [17]. One of the most important hallmarks of the complex structure is the positioning of the N-terminal portion of the Grb14 (BPS) (^376^LVAMDF) in the substrate binding groove of IRβ as a pseudosubstrate. This observation indicates that the Grb14 (BPS) is not only responsible for binding to the IR, but also functions as a competitive inhibitor for the tyrosine kinase activity of IRβ.

Considering the accumulating evidence for the pathogenicity of Grb14, together with the important role of the Grb14 (BPS) in binding to IRβ, compounds that inhibit the complex formation between the Grb14 (BPS) and IRβ are expected to have the potential to affect insulin signaling. In other words, compounds that interfere with the Grb14 function could lead to the development of drugs that improve insulin sensitivity and the duration of effect. In this context, the X-ray crystal structure of IRβ in complex with the Grb14 (BPS) was anticipated to provide valuable information for in silico SBDS. Indeed, Gondoin et al. recently identified a compound capable of inhibiting the protein–protein interaction (PPI) between Grb14 and IR [18]. They experimentally validated 1000 candidate compounds selected by virtual screening targeted to the whole structure of the IRβ–Grb14 (BPS) complex and found one active compound called C8 by bioluminescence resonance energy transfer and co-immunoprecipitation (Co-IP) experiments. In the present study, small compounds targeting the groove in IRβ for Grb14 (BPS) binding were explored by a highly efficient hierarchical in silico SBDS strategy composed of grid docking-based and genetic algorithm-based programs. Only ten top-ranked candidates were subjected to the experimental validation phase and action mechanism of one compound, which showed promising activity that was estimated by molecular dynamics (MD) simulation.

## 2. Results

### 2.1. In Silico Identification of Small Compounds Inhibiting Grb14-IRβ Complex Formation

**Figure 1:**
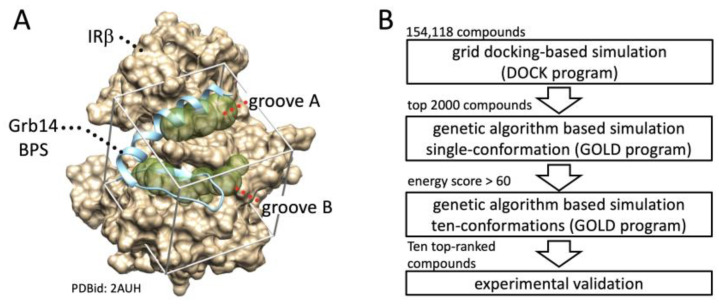
Schematic representation of in silico SBDS. (**A**). Binding pocket structures for in silico SBDS were prepared based on the crystal structure of IRβ in complex with part of Grb14 (BPS) (PDBid: 2AUH). The beige cloud represents the van der Waals molecular surface of IRβ. The cyan ribbon diagram represents Grb14 (BPS) (residues 373–409). The diaphanous sphere clusters, referred to as the binding groove A and B, were defined as the binding pockets for identification of novel inhibitor targeting PPI between IRβ and Grb14. (**B**). Schematic presentation of in silico SBDS. Ten compounds were screened from the compound library through DOCK-GOLD programs combined screening.

First, in silico SBDS was performed to search for compounds that interfere with the PPI between the IRβ and Grb14 (BPS). In general, the removal of the inhibitor structure from the protein–inhibitor complex provides a favorable binding pocket when identifying competitive inhibitors through in silico SBDS [19]. Herein, two binding pockets, shown as groove A and B, respectively, were identified after removal of the Grb14 (BPS) structure from 2AUH (Figure 1A). As shown in Figure 1B, the hierarchical docking simulations were performed by a high-throughput sorting based on grid docking (UCFS DOCK program), followed by a genetic algorithm-based sorting (GOLD program) for single and multiple (ten) conformations. Ten compounds with an average GOLD score of 68.0 or higher were eventually selected and purchased (Table S1 and Figure S1). If a compound with the desired biological activity is included among the ten candidates, it can be indicative of a high hit rate. Hereafter, the selected compounds are referred to as compounds **1**–**10**.

### 2.2. Experimental Validation of the Effect of Selected Compounds on IRS-1 Activity

**Figure 2:**
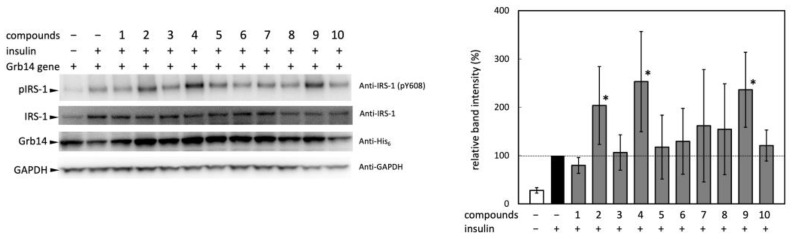
Effect of compounds **1**–**10** on insulin-stimulated IRS-1 activation. (**Left panel**), Activation of IRS-1 was evaluated as phosphorylation of Tyr608. HEK293T cells expressing Grb14 and IR were treated with 250 µg/mL compounds for 3 h and then stimulated with 1 mM insulin for 10 min. GAPDH was used as loading control. (**Right panel**), The band intensity of phosphorylated IRS-1: Quantifications of band intensity were performed in triplicate; *, *p* < 0.05.

Next, the selected ten compounds underwent experimental validation to assess their effects on insulin signaling at the cellular level. Grb14 has been reported to protect the phosphorylation status of the tyrosine residues in the activation loop of IRβ from dephosphorylation by protein tyrosine phosphatase 1B [9,10]; i.e., there was a concern that the phosphorylation state of the activation loop of IRβ may not directly reflect the intensity of the insulin signaling. Hence, the effect of the compounds on intracellular insulin signaling was assessed, not by tyrosine phosphorylation in the activation loop, but rather by that of IRS-1, which is located downstream of the receptor. The effect of the ten candidate compounds on the IRS-1 activation in HEK293T cells was evaluated by phosphorylation at Tyr608 in IRS-1 (Figure 2).

No significant difference was observed in the expression levels of IRS-1 and Grb14, though insulin-induced IRS-1 phosphorylation was significantly enhanced by the pretreatment with compounds **2**, **4**, and **9**, suggesting that these compounds facilitated downstream signal transduction of the IR. Due to considerable variability in band intensity, and an inability to identify significant differences except for the three compounds, these were selected for use in the subsequent animal experiment. While it was unclear whether the three compounds that enhanced insulin signaling bind to IRβ, they can be deemed to have a high hit rate in terms of demonstrating the anticipated biological activity.

### 2.3. Effects of Three Candidate Compounds on Blood Glucose Levels

**Figure 3:**
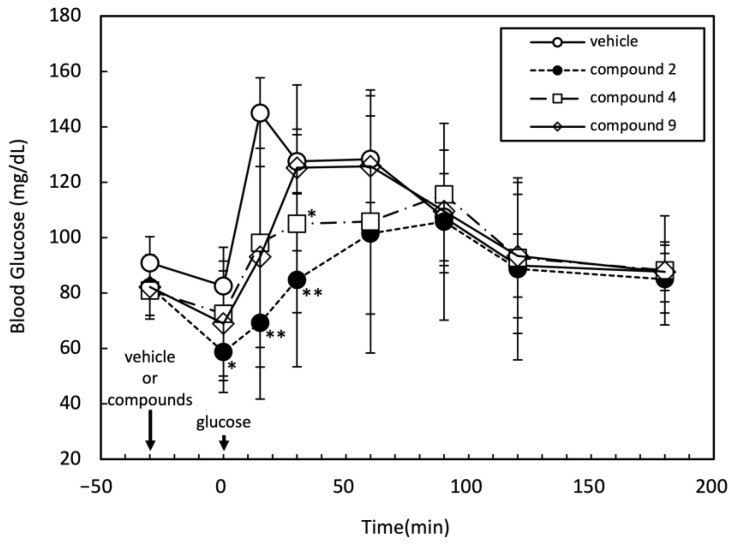
Effects of compounds **2**, **4**, and **9** on OGTT in rats. F344/Jcl rats (six weeks age, male, BW 112 ± 13 g, *n* = 5 in each group) were given intraperitoneal injection of vehicle alone (50% dimethyl sulfoxide (DMSO)), compounds **2**, **4** (1.6 mg/100 g BW) or **9** (0.8 mg/100 g BW) after overnight fasting. Thirty minutes after injection, rats were given oral glucose administration (2 g/kg BW). *, *p* < 0.05; **, *p* < 0.01.

To assess whether the compounds **2**, **4**, and **9**, which were suggested to promote insulin signaling, function in vivo, their effects on blood glucose levels in rats were evaluated through an oral glucose tolerance test (OGTT). After intraperitoneal injection of the medication, glucose was administered orally 30 min thereafter. During the experiment, the time course of the blood glucose level was monitored, as shown in Figure 3. Among the tested three compounds, compound **2** significantly suppressed the increase in the blood glucose level at 0, 15, and 30 min after oral glucose administration. Compound **4** also showed a moderate but significant reduction in the blood glucose level 30 min after oral glucose administration. At least compound **2** was suggested to affect the insulin signaling in vivo.

### 2.4. Effect of Compound **2** on IRβ–Grb14 Complex Formation

**Figure 4:**
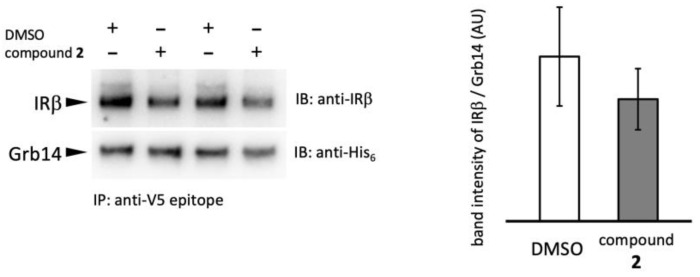
Effect of compound **2** on PPI between IRβ and Grb14. (**Left panel**), Co-precipitation of IRβ (**upper**) with agarose beads bound to Grb14 (**lower**). Grb14 and IRβ were incubated with compound **2** (200 µg/mL) or 2% DMSO at 4 °C overnight, then washed and visualized by immunoblotting. (**Right panel**), The ratio of band intensity of IRβ/Grb14: Quantifications of band intensity were performed in duplicate.

To determine whether compound **2** affects the PPI between IRβ and Grb14, a Co-IP experiment was attempted (Figure 4). Grb14 was immobilized on agarose beads via a V5 epitope tag; then, WGA purified IR was added with compound **2** or 2% DMSO (negative control) [18]. The IRβ–Grb14 complex formation was evaluated by a band intensity ratio of co-precipitated IRβ and beads-bound Grb14. The compound **2** slightly reduced the band intensity, representing the formation of the IRβ–Grb14 complex at a concentration of 200 µg/mL; no significant difference was observed compared to the control, and complete inhibition of complex formation did not occur. The results suggest that compound **2** is unable to completely disrupt the formation of the IRβ–Grb14 complex.

### 2.5. Predicted Protein–Inhibitor Interaction by MD Simulation

**Figure 5:**
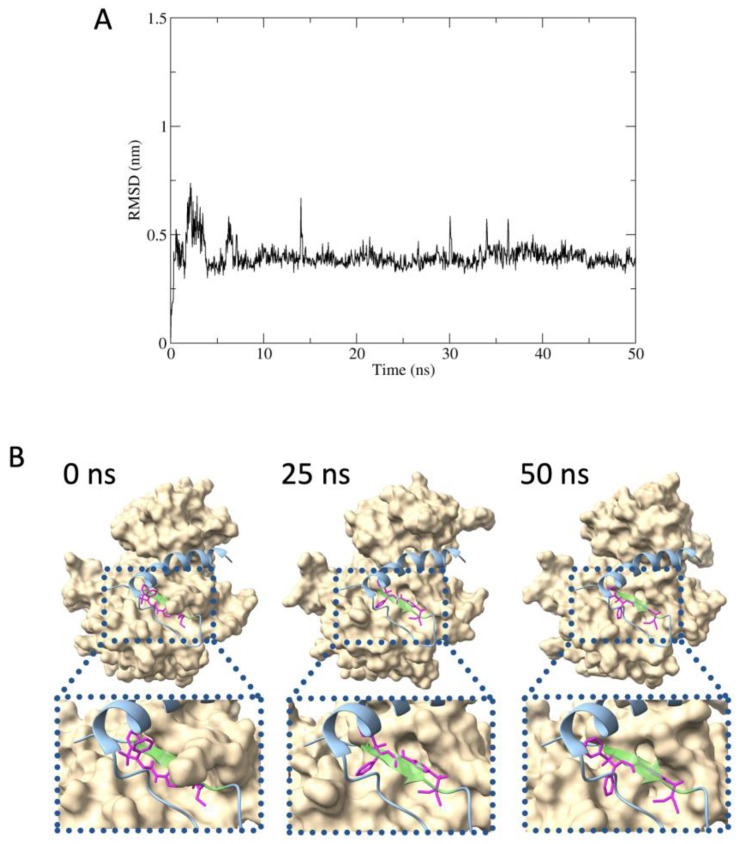
MD-simulated interference of physical contact between IRβ and Grb14 (BPS) by compound **2**. (**A**), The RMSD plots for the IRβ and compound **2** complex during 50 ns timescale. (**B**), Structure of IRb–compound **2** at 0, 25, and 50 ns in the MD simulation, superimposed with Grb14 (BPS). IRβ, compound **2**, and Grb14 (BPS) are represented by surface (beige), stickle (magenta), and ribbon (cyan) models, respectively. The light green portion in Grb14 (BPS) indicates the pseudosubstrate region for IRβ tyrosine kinase.

The MD simulations were conducted to assess the binding stability of compound **2** with IRβ to estimate the inhibitory mechanism of compound **2** on Grb14 function: (i) examining the stability of the complex formed by IRβ and compound **2** over the 50-nanosecond timescale; (ii) confirming whether the pseudosubstrate portion within Grb14 (BPS) and compound **2** competitively bind to IRβ. The root mean square deviation (RMSD) between compound **2** to IRβ maintained around 0.4 nm during the simulation; stable binding of compound **2** with IRβ was suggested (Figure 5A). According to the molecular mechanics Poisson–Boltzmann surface area (MM-PBSA) analysis, the binding free energy was calculated to be −26.46 kcal/mol. Figure 5B shows the estimated structures of IRβ in complex with compound **2** at 0, 25, and 50 nanoseconds in the MD simulation, overlaid with the Grb14 (BPS). The binding of compound **2** to the tyrosine kinase pseudosubstrate region within the BPS region was consistently observed (residues ^376^LVAMDF^381^, represented in light green).

## 3. Discussion

Compounds that intervene at PPIs occurring downstream of the insulin receptor have a mechanism of action distinct from existing drugs that modulate circulating insulin levels {e.g., sulfonylurea, dipeptidyl peptidase-4 (DPP4) inhibitors, glucagon-like peptide-1 receptor (GLP1-R) agonist, sodium-glucose cotransporter 2 (SGLT2) inhibitors} [20]. Development of compounds that intervene at PPIs occurring downstream of the insulin receptor would be beneficial in understanding insulin resistance. In this context, in silico SBDS has been anticipated as an efficient technique. Since the Grb14-mediated insulin signal suppression is based on the PPI between Grb14 (BPS) and IRβ, the structural data of 2AUH were employed for the virtual compound screening. In general, to screen for a competitive inhibitor through virtual screening, the utilization of the apo-form structure is sometimes inappropriate. The structural data of IRβ in 2AUH were expected to provide preferable binding pockets or groove structures after removing the Grb14 (BPS) structure. The combination of high-throughput screening by the grid docking (DOCK program) and the precise evaluation of binding characteristics by the genetic algorism (GOLD program) enables efficient compound screening and reduces computational costs. Three of ten compounds, selected from 154,118 compounds, were found to promote IRS-1 activation, and subsequent in vivo experiments suggested that one of them (compound **2**) inhibited the increase in blood glucose levels caused by glucose loading. The combined computational and experimental approach performed in this study has shown the potential to identify compounds that target PPIs without having to test a large number of compounds.

With respect to the structural data of 2AUH, Depetris et al. observed that Leu376 in the pseudosubstrate within the Grb14 (BPS) occupies the kinase active site in IRβ, displacing a substrate tyrosine. Additionally, Val377, Met379, and Phe382 in the pseudosubstrate portion are involved in the interaction with Leu1171, Gly1169, and Gly1167 in IRβ, respectively [17]. The MD simulations predict that compound **2** binds to IRβ competitively with the pseudosubstrate portion during 50 ns, and ligand interaction (LI) predictions indicate that compound **2** forms hydrogen bonds with Gly1166 and Leu1171, and cation–pi interaction with Gly1167 in IRβ (Figure S2). In addition to these specific bindings, hydrophobic interactions toward the groove B were also predicted. Whereas the present Co-IP analysis showed co-precipitation of IR was not effectively suppressed even at a high concentration of compound **2**, suggesting compound **2** could not abolish the formation of the IRβ–Grb14 complex. In the design of molecularly targeted drugs, compound structures are designed for specific binding sites on the protein structure, such as substrate binding pockets. However, when targeting PPI, it appears necessary to target a broader area, such as the interaction surfaces [21]. In addition, although the BPS region is known as the primary region responsible for binding to IRβ, the SH2 domain adjacent to the C-terminal side of the BPS region is also known to assist in the binding [8,15,17]. From these perspectives, it would be difficult to eliminate the interaction between Grb14 and IRβ, which occurs on the surfaces of proteins and at multiple interaction sites with small molecules. Nonetheless, the possibility that compound **2** may exert its effects on a target distinct from the IRβ–Grb14 complex formation cannot be excluded; more detailed studies are needed to elucidate the mechanism of action of compound **2**. Furthermore, it is assumed that compound **2** obtained from the compound library is not a natural product. It is important to use compound **2** as a seed compound in future structure–function relationship (SAR) studies to analyze the pharmacophores that play an important role in the biological activity.

In general, during high-throughput screening, the discovery rate of hit compounds is relatively low, typically less than 1% in most cases [22]. This necessitates the utilization of extensive compound libraries to generate a sufficient number of hit compounds, facilitating the progression of drug development programs. The substantial size of these libraries, coupled with the extended lead times of campaigns, contribute significantly to the overall costs associated with the screening process [23]. In this study, compound screening was conducted on a scale exceeding 150,000 compounds, and experimental investigations were performed on 10 selected compounds, among which, one exhibited biological activity. These results strongly suggest that in silico SBDS composed of the DOCK and GOLD programs could contribute to cost and labor efficiency in the screening of compounds that interfere with PPI. In conclusion, the efficient exploration of small compounds impacting insulin signaling was achieved through the combination of in silico computational chemistry and experimental validation. The integration of these approaches proves to be effective in the search for compounds affecting insulin signaling pathways.

## 4. Materials and Methods

### 4.1. Materials

The candidate compounds identified through SBDS were purchased from ChemBridge (San Diego, CA, USA). Gene constructs for expression of Grb14 (hGrb14/pcDNA3.1D/V5-His-TOPO) and IR (pFN21AE3464) in mammalian cells are reported previously [24]. Rabbit anti-IRS-1 and anti-phospho-IRS-1 (Tyr608) mouse/(Tyr612) human antibodies were purchased from Merck (Whitehouse Station, NJ, USA). Horseradish peroxidase (HRP)-conjugated anti-His-tag monoclonal antibody was purchased from MLB (Nagoya, Japan). HRP-conjugated anti-glyceraldehyde-3-phosphate dehydrogenase (GAPDH) monoclonal antibody was purchased from FUJIFILM Wako Pure Chemical Industries (Osaka, Japan). HRP-conjugated anti-IRβ mouse monoclonal antibody and goat HRP-conjugated anti-rabbit IgG were obtained from Santa Cruz Biotechnology (Santa Cruz, CA, USA). HEK293T(CRL-3216) and COS-7(CRL-1651) were purchased from American Type Culture Collection (Manassas, VA, USA). Other chemicals of reagent grade were obtained from Sigma, FUJIFILM Wako Pure Chemical Industries, and Nacalai Tesque (Kyoto, Japan).

### 4.2. In Silico Structure-Based Drug Screening

Preparation of the virtual chemical library composed of 154,118 compounds was reported previously [19]. Structural data of the IRβ in complex with the Grb14 (BPS) region (PDB-id: 2AUH) were used after removal of the BPS region structure. Protonation states and partial charges were adjusted at pH 7.0 using the Protonate 3D module in the molecular operating environment (MOE 2019) (Chemical Computing Group, Montreal, QC, Canada). The screening system, constructed using UCSF DOCK (version 6.3) and CCDC GOLD suite (version 5.3), was employed to search for inhibitors targeting the IRβ–Grb14 complex formation. Throughout this study, these programs are referred to as the DOCK and GOLD programs, respectively. Initially, high-throughput screening was conducted using the DOCK program, based on grid docking, to estimate potential binding affinities. The top 2000 compounds were selected based on this screening. Subsequently, compounds with a GOLD score greater than 60 for a single compound conformation were identified using the GOLD program. To generate diverse conformations for each compound, the Conformation Search module in MOE was utilized, resulting in ten distinct stable conformations for each compound. These conformations then underwent a rescreening process using the GOLD program. The obtained average GOLD scores for the selected compounds were then compared. Finally, the top ten compounds were purchased from ChemBridge and subjected to experimental validation. The precise molecular binding interactions between the compounds and the amino acids forming the IRβ substrate-binding cavity were inferred using the LI and PLIF modules within the MOE software.

### 4.3. Phosphorylation of IRS-1

HEK293T cells (10^5^ cells/well) were seeded into 24-well plates 12 h before cDNA transfection and were cultured in high-glucose Dulbecco’s modified Eagle medium (DMEM) supplemented with 10% fetal bovine serum (FBS), 100 U/mL penicillin, 100 µg/mL streptomycin, and 4 mM l-glutamine at 37 °C in a humidified atmosphere containing 5% CO_2_. The expression vectors for human IR (0.2 µg) and Grb14 vectors (0.8 µg) were co-transfected using X-tremeGENE 9 (Roche Diagnostics, Basel, Switzerland) transfection reagent. The cell culture was continued for 48 h, then medium was replaced with serum-deprived DMEM. Incubation was continued for 3 h after the addition of the test compounds (30 µg/mL), followed by treatment with 1 µM insulin for 10 min. The cultures were rinsed twice with DPBS and lysed with lysis buffer: 50 mM Tris-HCl (pH 7.4), 150 mM NaCl, 1% Triton X-100, 5 mM EDTA, 50 mM NaF, 1 mM orthovanadate, 30 mM sodium pyrophosphate, and Complete Ultra, Mini, EDTA-free (Roche Diagnostics). The cells were sonicated and centrifuged at 13,000× *g* at 4 °C for 20 min, and the supernatants were subjected to immunoblot analysis. HRP was visualized by Chemi-Lumi One L (Nacalai tesque) and analyzed with LuminoGraph I (ATTO Corporation, Tokyo, Japan). GAPDH was used as loading control.

### 4.4. Oral Glucose Tolerance Test

An OGTT was performed in 6-week-old male Fischer (F344/Jcl) rats (six weeks age, male, BW 112 ± 13 g, *n* = 5 in each group) purchased from CLEA Japan, Inc. (Tokyo, Japan). The rats were fasted overnight followed by intraperitoneal injection of vehicle or Grb14 inhibitors dissolved in 50% DMSO. DMSO was administered at a dosage of 0.5 mL/kg body weight. The reported LD_50_ of DMSO in rats is 9.9 mL/kg body weight (i.p.) [25]. Considering that the administered amount of DMSO was significantly below the LD_50_, there are no concerns regarding the toxicity of DMSO to rats. Thirty minutes after injection, rats were given glucose orally (2 g/kg BW). Blood glucose levels were measured from tail vein at 0, 15, 30, 60, 90, 120, 150, and 210 min after the injection of vehicle or Grb14 inhibitors.

### 4.5. Co-Immunoprecipitation (Co-IP)

The Co-IP experiment was conducted according to the previously reported method with minor modifications [18,24]. In brief, Grb14 with V5 epitope and his-tags on the C-terminal, and human IR were separately overexpressed in COS-7 cell cultures. The cell cultures were maintained overnight in a serum-free medium and then stimulated with 1 µM insulin for 10 min. After rinsing with PBS, the cells were treated with the same lysis buffer used in the experiments evaluating IRS-1 phosphorylation. The supernatants containing IR or Grb14 were collected through brief sonication and centrifugation (13,000× *g* at 4 °C for 20 min). The supernatant containing Grb14 was incubated with agarose-conjugated Anti-V5-Tag beads (FUJIFILM Wako) for 12 h at 4 °C. After washing the beads three times with the lysis buffer, IR, purified with the Glycoprotein Isolation Kit (Thermo Fisher Scientific, Waltham, MA, USA) was added together with 2% DMSO (as negative control) or compound **2** (200 µg/mL) and then incubated at 4 °C overnight with gentle agitation. After washing the beads with the lysis buffer three times, proteins bound to the beads were analyzed by Western blotting, as described above.

### 4.6. Molecular Dynamics Simulation

MD simulations were performed using the GROMACS package, with slight modifications to the previously reported methods [26]. In brief, the simulation system was prepared with the CHARMM-GUI web server, where TIP3P was used as a water molecule. The cutoff value of 1.2 nm was used as an interatomic distance for the van der Waals force and electrostatic interaction. The particle mesh Ewald method was used to calculate long-range electrostatic interactions. The LINCS constraint algorithm was used for the energy minimization, equilibration, and production MD calculations. Energy minimization calculations were conducted in up to 5000 steps using the steepest descent algorithm. The equilibration calculations were conducted in one step under the NVT conditions (310.00 K), followed by two steps under NPT conditions (310.00 K, 1 bar). Finally, 50 ns production MD calculations were performed with a time step of 2 fs. MD trajectories were analyzed using g_rms in the GROMACS package.

## Data Availability

The data presented in this study are available upon request from the corresponding author.

## Supplementary Materials

The following Supporting Information can be downloaded at: https://www.mdpi.com/article/10.3390/molecules29010198/s1, Table S1: Ten candidate compounds identified by in silico SBDS; Figure S1: Chemical structures of ten candidate compounds identified by in silico SBDS; Figure S2: Ligand interaction (LI) between IRβ and compound **2** complex formation.
