# Supplementary material for: Computational Screening and Experimental Validation of Inhibitor Targeting the Complex Formation of Grb14 and Insulin Receptor
Source: Molecules. 2023 Dec 29;29(1):198. doi: 10.3390/molecules29010198 (PMC10780909; doi:10.3390/molecules29010198)
Supplement: Supplementary file 1 [file molecules-29-00198-s001.zip › molecules-2787441-supplementary.pdf]

Table S1. Ten candidate compounds identified by in silico SBDS.

compound	ChemBridge IDs	IUPAC name	M.W.	cLogP	tPSA	GOLD score
**1**	7389795	4-methoxy-*N*-(4-{[(2-phenylethyl)amino]sulfonyl}phenyl)benzenesulfonamide	447	3.86	102	75.9
**2**	7788851	*N*[1]-{4-[(diethylamino)sulfonyl]phenyl}-*N*[2]-phenyl-*N*[2]-(phenylsulfonyl)glycinamide	502	4.46	104	75.8
**3**	7780058	2-({3-[(2-ethoxybenzoyl)amino]benzoyl}amino)benzoic acid	404	4.70	105	72.4
**4**	7972614	*N,N′*-bis(4-methoxybenzyl)-1-[(4-methylphenyl)sulfonyl]-1H-1,2,4-triazole-3,5-diamine	494	3.18	107	71.1
**5**	6824862	*N*-(1,5-dimethyl-3-oxo-2-phenyl-2,3-dihydro-1H-pyrazol-4-yl)-2-{[5-(phenoxymethyl)-4-phenyl-4H-1,2,4-triazol-3-yl]thio}acetamide	527	1.42	96	70.8
**6**	7968391	*N*-1,3-benzothiazol-2-yl-2-({5-[3-(4-methoxy-2-methylphenyl)propyl]-4-methyl-4H-1,2,4-triazol-3-yl}thio)acetamide	468	4.31	82	70.4
**7**	6945187	3-[4-(4-bromophenyl)-1,3-thiazol-2-yl]-7-hydroxy-8-{[4-(2-hydroxyethyl)-1-piperazinyl]methyl}-2H-chromen-2-one	542	4.92	90	68.8
**8**	7355746	*N*-(2,4-dichlorobenzyl)-*N*-[(4-methylphenyl)sulfonyl]glycine	388	4.86	75	68.3
**9**	7917692	*N*-[(1-ethyl-2-oxo-1,2-dihydrobenzo[cd]indol-6-yl)sulfonyl]tryptophan	464	3.26	120	68.1
**10**	6082385	4-{4-[(phenoxyacetyl)amino]phenoxy}phthalic acid	407	3.58	122	68.0

1

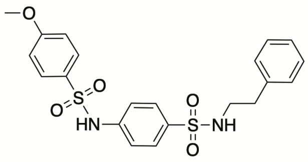

2

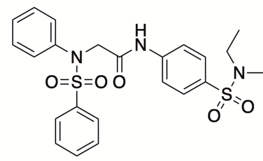

3

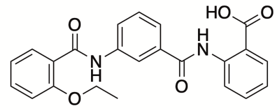

4

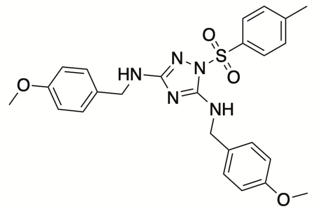

5

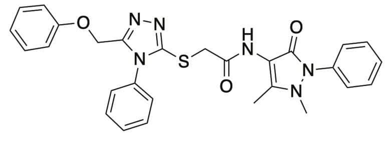

6

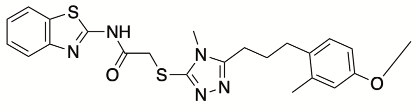

7

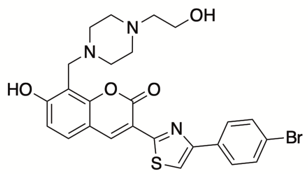

8

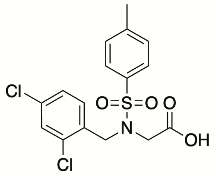

9

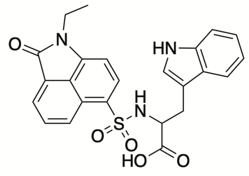

10

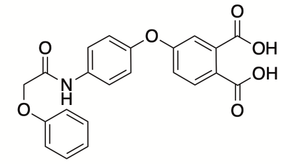

Figure S1. Chemical structures of ten candidate compounds (**1-10**) identified by in silico SBDS.

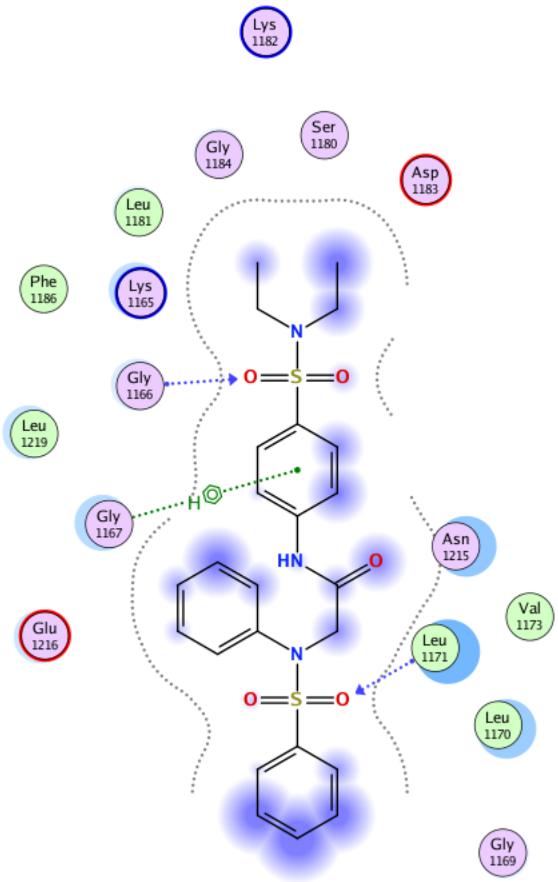

Figure S2. Ligand Interaction (LI) between IRβ and compound **2.** The data shows predicted interaction at 50 ns in the MD simulation. The dotted blue allows and green line represent hydrogen bonds and cation-π interaction, respectively. The blue crowds on compound **2** indicate hydrophobic interactions with the binding pocket.
